# Supplementary material for: Identification of Fucosylated SERPINA1 as a Novel Plasma Marker for Pancreatic Cancer Using Lectin Affinity Capture Coupled with iTRAQ-Based Quantitative Glycoproteomics
Source: Int J Mol Sci. 2021 Jun 4;22(11):6079. doi: 10.3390/ijms22116079 (PMC8200073; doi:10.3390/ijms22116079)
Supplement: Supplementary file 1 [file ijms-22-06079-s001.zip › Supplementary Files/Supplementary figures.pptx]

## Slide 1
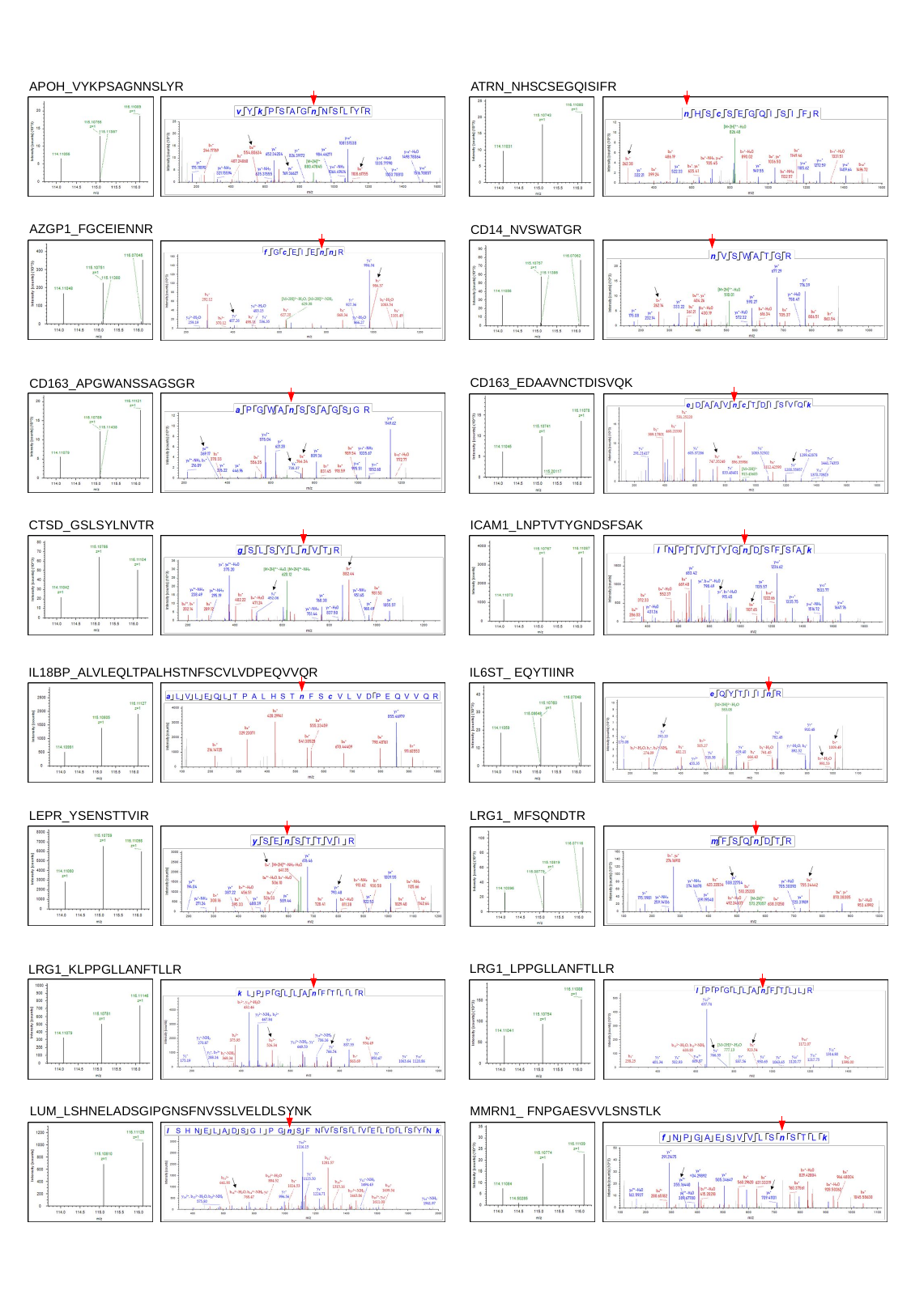

APOH_VYKPSAGNNSLYR
ATRN_NHSCSEGQISIFR
AZGP1_FGCEIENNR
CD14_NVSWATGR
CD163_EDAAVNCTDISVQK
CD163_APGWANSSAGSGR
ICAM1_LNPTVTYGNDSFSAK
CTSD_GSLSYLNVTR
IL18BP_ALVLEQLTPALHSTNFSCVLVDPEQVVQR
IL6ST_ EQYTIINR
LEPR_YSENSTTVIR
LRG1_ MFSQNDTR
LRG1_LPPGLLANFTLLR
LRG1_KLPPGLLANFTLLR
MMRN1_ FNPGAESVVLSNSTLK
LUM_LSHNELADSGIPGNSFNVSSLVELDLSYNK

## Slide 2
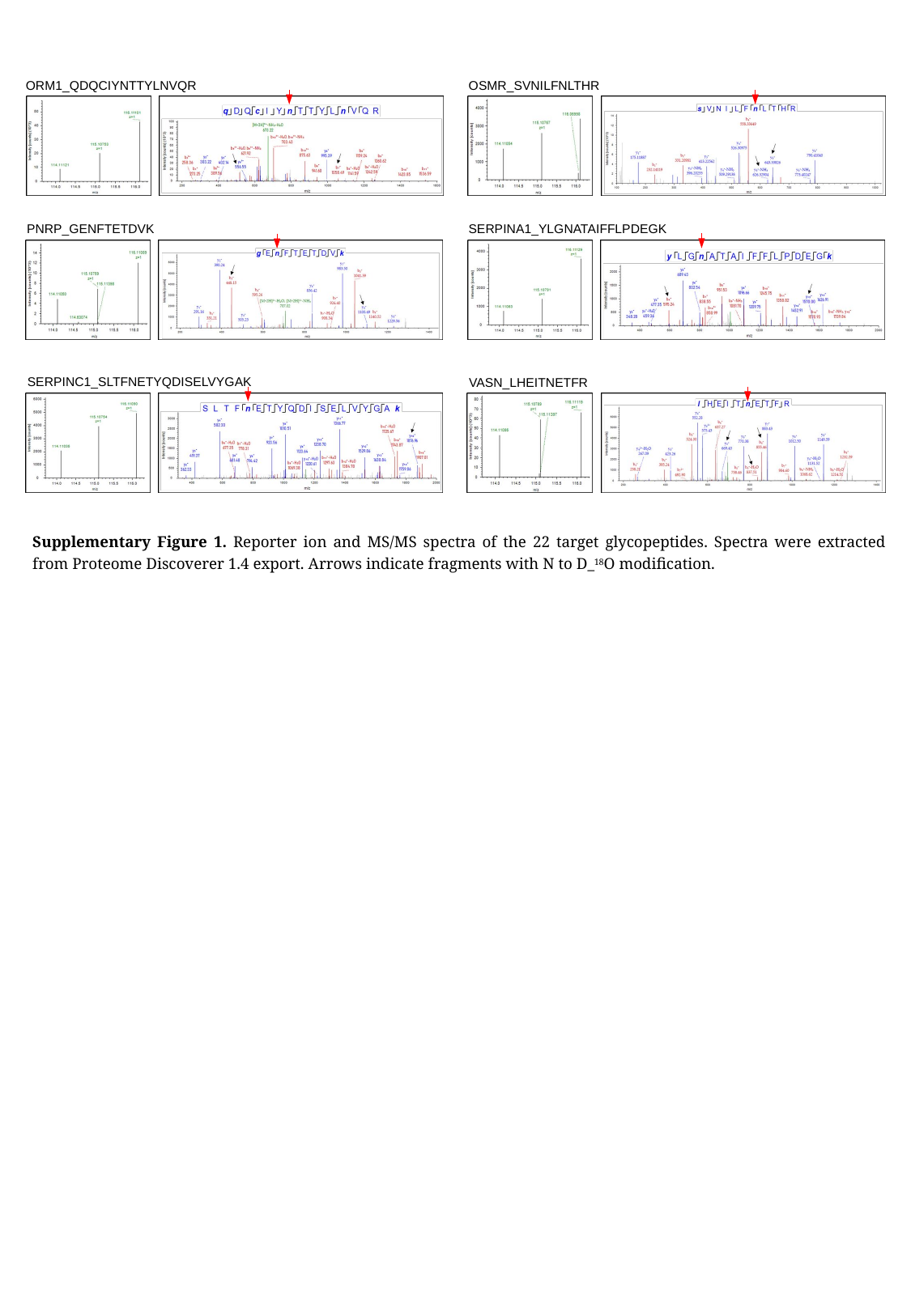

OSMR_SVNILFNLTHR
ORM1_QDQCIYNTTYLNVQR
PNRP_GENFTETDVK
SERPINA1_YLGNATAIFFLPDEGK
SERPINC1_SLTFNETYQDISELVYGAK
VASN_LHEITNETFR
Supplementary Figure 1. Reporter ion and MS/MS spectra of the 22 target glycopeptides. Spectra were extracted from Proteome Discoverer 1.4 export. Arrows indicate fragments with N to D_18O modification.

## Slide 3
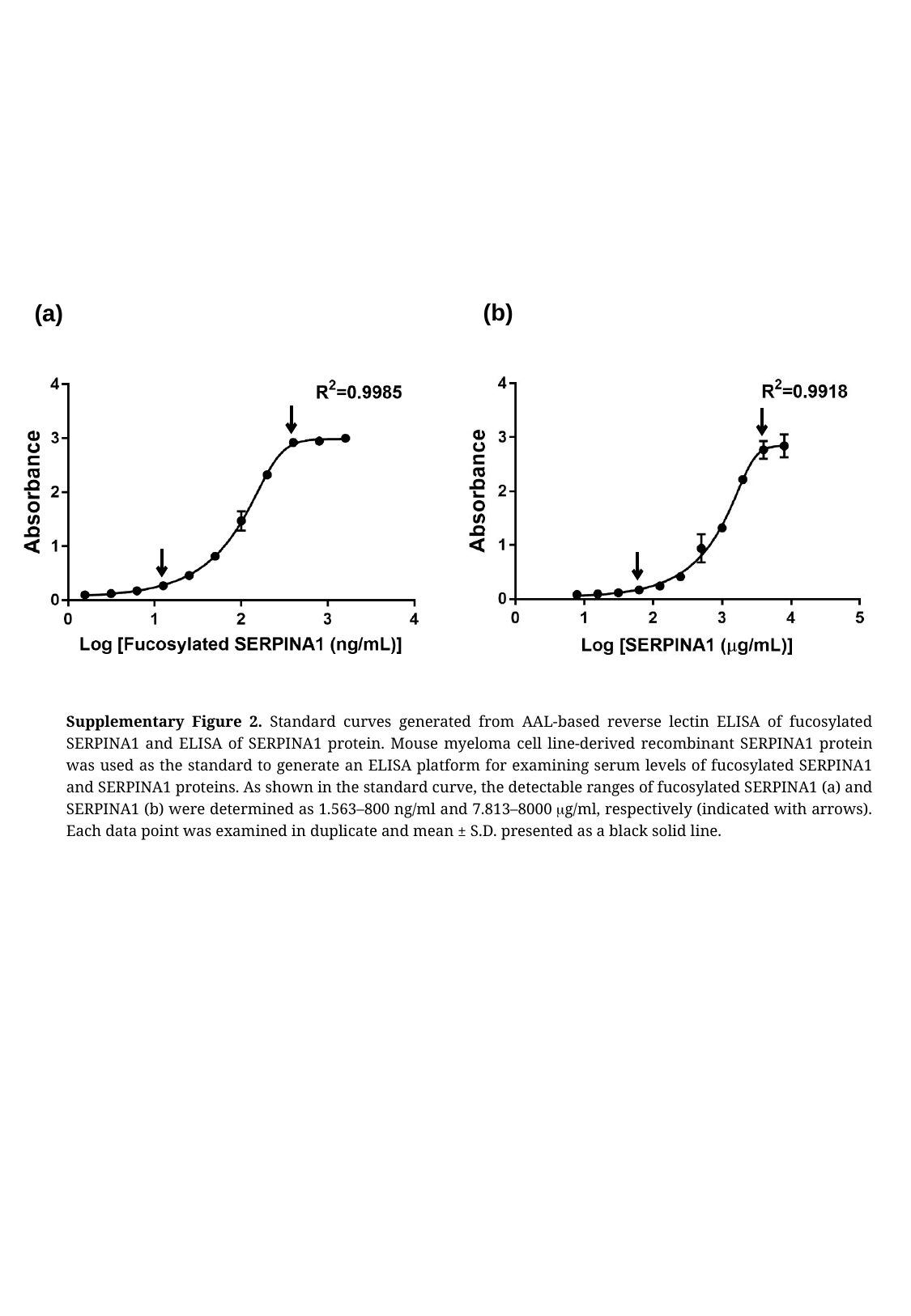

(b)
(a)
Supplementary Figure 2. Standard curves generated from AAL-based reverse lectin ELISA of fucosylated SERPINA1 and ELISA of SERPINA1 protein. Mouse myeloma cell line-derived recombinant SERPINA1 protein was used as the standard to generate an ELISA platform for examining serum levels of fucosylated SERPINA1 and SERPINA1 proteins. As shown in the standard curve, the detectable ranges of fucosylated SERPINA1 (a) and SERPINA1 (b) were determined as 1.563–800 ng/ml and 7.813–8000 mg/ml, respectively (indicated with arrows). Each data point was examined in duplicate and mean ± S.D. presented as a black solid line.
